# Shallow and deep learning classifiers in medical image analysis

**DOI:** 10.1186/s41747-024-00428-2

**Published:** 2024-03-05

**Authors:** Francesco Prinzi, Tiziana Currieri, Salvatore Gaglio, Salvatore Vitabile

**Affiliations:** 1 Department of Biomedicine, Neuroscience and Advanced Diagnostics (BiND), University of Palermo, Palermo, Italy; 2 Department of Computer Science and Technology, University of Cambridge, Cambridge, CB2 1TN UK; 3 Department of Engineering, University of Palermo, Palermo, Italy; 4 Institute for High-Performance Computing and Networking, National Research Council (ICAR-CNR), Palermo, Italy

**Keywords:** Artificial intelligence, Deep learning, Explainable AI, Machine learning classifiers, Shallow learning

## Abstract

An increasingly strong connection between artificial intelligence and medicine has enabled the development of predictive models capable of supporting physicians’ decision-making. Artificial intelligence encompasses much more than machine learning, which nevertheless is its most cited and used sub-branch in the last decade. Since most clinical problems can be modeled through machine learning classifiers, it is essential to discuss their main elements. This review aims to give primary educational insights on the most accessible and widely employed classifiers in radiology field, distinguishing between “shallow” learning (*i.e.,* traditional machine learning) algorithms, including support vector machines, random forest and XGBoost, and “deep” learning architectures including convolutional neural networks and vision transformers. In addition, the paper outlines the key steps for classifiers training and highlights the differences between the most common algorithms and architectures. Although the choice of an algorithm depends on the task and dataset dealing with, general guidelines for classifier selection are proposed in relation to task analysis, dataset size, explainability requirements, and available computing resources. Considering the enormous interest in these innovative models and architectures, the problem of machine learning algorithms interpretability is finally discussed, providing a future perspective on trustworthy artificial intelligence.

**Relevance statement** The growing synergy between artificial intelligence and medicine fosters predictive models aiding physicians. Machine learning classifiers, from shallow learning to deep learning, are offering crucial insights for the development of clinical decision support systems in healthcare. Explainability is a key feature of models that leads systems toward integration into clinical practice.

**Key points**

• Training a shallow classifier requires extracting disease-related features from region of interests (*e.g.,* radiomics).

• Deep classifiers implement automatic feature extraction and classification.

• The classifier selection is based on data and computational resources availability, task, and explanation needs.

**Graphical Abstract:**

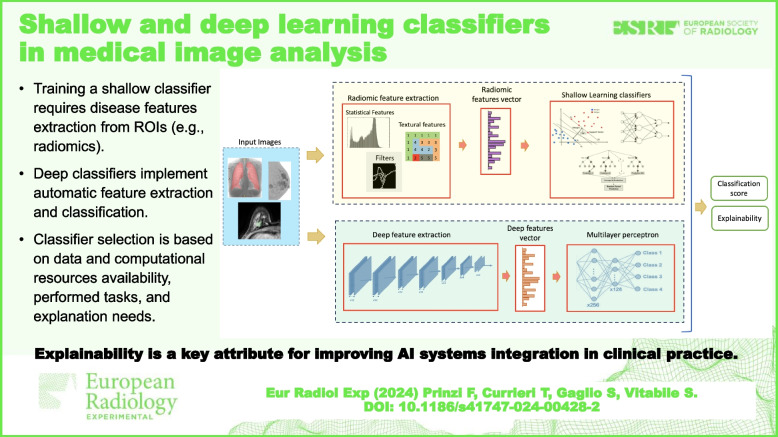

## Background

A large part of the main machine learning (ML) applications in medicine concerns the analysis of radiological images. Remarkable applications include breast cancer detection [1], cardiac disease diagnosis [2], prognostication of treatment responses [3], and numerous other scenarios [4, 5]. ML is a subfield of artificial intelligence (AI) that includes the concepts of “shallow learning” (SL) and “deep learning” (DL).

The term SL is employed to categorize all algorithms that do not fall within the realm of deep learning architectures. Specifically, it encompasses traditional approaches and excludes advanced architectures that have the multilayer and hierarchical structure of deep networks. It was recently said that SL refers to most ML models proposed prior to 2006, including the so-called shallow neural networks (neural networks with only one hidden layer), linear regression, logistic regression (LR), support vector machines (SVM), decision trees (DT), and k-nearest neighbors [6]. For this reason, when we mention SL, we are referring to the previously mentioned methods (SVM, DT, LR, etc.), with the exclusion of deep architectures such as convolutional neural networks (CNNs) and transformers. DL methods are defined as all deep architectures, such as neural networks (NN) with many layers, including CNNs, vision transformers (ViTs), recurrent NNs, restricted Boltzmann machines, deep belief networks, and many other architectures [7].

Classification problems aim to predict the category (class) to which a given input belongs and typically fall within the domain of supervised learning. The input could be a normal or abnormal tissue, a vessel, a tumor, etc. In addition, training a classifier necessitates a reference standard (also called “ground truth”), such as a histological examination, the response to a particular treatment, or a well-established event that represent the class required for supervised learning. In the analysis of radiological images, the regions of interest (ROI), *i.e.,* the inputs to be classified, need to be represented in salient and informative form. This crucial step is performed through a process called feature extraction, which can be executed by means of two distinct methodologies: “handcrafted” [8, 9] and “deep” [10] feature extraction.

The decision between these approaches strongly influences the selection of a classifier, determining whether a deep or shallow classifier is more appropriate. Despite the proven ability to develop high-performance models to support the physician’s decision-making process, training these algorithms is overly complex and hides many pitfalls. Feature extraction, feature selection, training, and model validation are all steps that need to be addressed with high accuracy and robustness [11].

This review aims to give primary educational insights on the most accessible and widely employed classifiers in radiology field discussing the following:

- The main concepts related to the most widely used ML classifiers in the literature and their training
- The main differences between shallow and deep learning classifiers, including the methods and the related feature extraction processes involved
- Some practical guidelines on how to choose a classifier, focusing mainly on data and computational resource availability, the task, and explainability requirements
- The importance of explainable AI for the actual integration of ML models in clinical practice

## Classifiers: main concepts

Classification tasks aim to assign a class label to instances described by their respective features. These numerical features serve as the *input data* and encapsulate information about the object being classified, such as the tumor’s shape, margins, density, the extent of vessel occlusion, vital parameter values, or the texture within a ROI, among other attributes. In certain DL architectures, the input can encompass only the ROI or the whole image.

The *output variable* corresponds to the label or class associated with each input data point. When this label represents a binary outcome, such as the presence or absence of a disease, the effectiveness or ineffectiveness of a therapy, or the benign or malignant nature of a lesion, the process is referred to as *binary classification*. Traditionally, this outcome is encoded or tokenized as *0* to indicate the negative class (representing the absence of disease, ineffectiveness of therapy, or benign nature of the lesion) or as *1* to denote the positive class (indicating the presence of disease, effectiveness of therapy, or malignancy of the lesion). When it has more than two classes, it is called *multiclass classification*. The presence of the target variable for each sample makes the classification algorithms belong to supervised learning algorithms, in which the target information guides training. The model is properly trained only when it makes correct predictions, or rather generalizes, on unknown data (*i.e.,* data that it has never seen during the training phase) [12].

To evaluate this generalization capability, the dataset is divided into *training*, *validation*, and *test sets*. The three sets are distinct, meaning that each data point can only be a part of one of the three subsets. While historically the terms validation and test set, particularly in medical literature, have been erroneously used interchangeably, the introduction of the CLEAR [13] and CLAIM [14] guidelines has provided clear definitions. In fact, the terms *training set* and *validation set* are used for the data partitions with which the algorithm is trained and tuned, respectively. The term *test set* is used for the data with which the model is verified internally or externally.

### Training, validation, and test steps

*Training data* are employed to learn a separating hyperplane or, in a broader sense, a function to make predictions about the class of unseen data points. This function has to be able to associate to each unseen input, the related label. *Validation data* are used to set the algorithm’s hyperparameters. The algorithm’s *hyperparameters* are the arguments required by the algorithms to improve the training process. Conversely, the parameters are the variables defining the function to separate the class. For example, for a NN, the parameters are the weights that identify the classification hyperplane; the hyperparameters, as an example, are the number of hidden layers, the number of neurons per layer, the activation functions, and the learning rate. The validation set is used to select the best model during the training process and choose the algorithm hyperparameters: the algorithm is trained with different hyperparameter configurations and “tested” with the validation data. In the end, the hyperparameters that provide the highest performance on the validation data are selected. This process is commonly called hyperparameters tuning. There are several methods for automatic hyperparameter tuning, recently accessible to nonprofessional users with limited computing expertise [15, 16]. After training the model with the best hyperparameters, it must be tested on the test set, *i.e.,* data not used during training and validation steps (unknown data). The purpose of this step is to evaluate the actual generalization capabilities of the trained model. If the model is unable to generalize to unseen data, *i.e.,* test data, then the model could be underfitting (a model is excessively simplistic and fails to capture complex patterns) or overfitting (a model fits training data too closely, resulting in poor generalization to new, unseen data) [17].

For applications involving datasets with several thousand samples, it is usual to partition the dataset into training, validation, and test subsets. The specific ratio for this division may vary, with common percentage splits being 70-10-20 or 70-15-15. However, there is no strict rule dictating the exact proportions. For classification problems, it is common to generate these subsets in a stratified manner, that is, to have in each subset a balanced/representative number of samples for each class. In scenarios where the dataset is composed of only a few hundred samples, a standard practice is to split it only into training and test sets. Subsequently, a *cross-validation strategy* is often employed exclusively in the training set. This cross-validation approach, consisting of dividing the training set into subsets (called folds), and in each round select one of these folds as the validation set and the others as the training set, is used for both training and fine-tuning the model, and, ultimately, the model’s performance is assessed on the dedicated test set [18]. In the case of very small datasets (about less than 100 samples), the leave-one-out method is typically employed [19, 20]. However, the leave-one-out is more susceptible to overfitting than k-fold cross-validation [21]. For this reason, a k-fold cross-validation is mainly adopted when more than 100 samples are available [22, 23].

## Shallow learning classifiers

Shallow learning, also known as “traditional ML,” refers to a class of algorithms that typically involve a limited number of layers or levels of abstraction in their models. To train the SL methods discussed in the next subsections, it is necessary to provide a feature vector as input. When dealing with medical images, this entails converting the image or the ROIs into features. This conversion can be achieved through either manual techniques, such as the *radiomics* workflow for handcrafted features extraction [24, 25] or by using deep architectures to extract learned features (or “deep features”).

### Logistic regression

Logistic regression is a technique used to identify the relationship between the dependent and independent variables. The dependent variable is the target class to be predicted. The independent variables are the attributes or features used to predict the target class [26]. Like other classifiers, it returns the probability that an instance belongs to a particular class. In LR, the separating function is commonly referred to as the logistic function (or sigmoid function). This function fits the curve to a group of points to minimize the error and compresses the output of a linear equation between 0 and 1. For the training process, a loss function called “maximum likelihood estimation” is used to estimate the error between the predicted and true output. If the estimated output for an instance is greater than 50%, it means the model predicts the positive class, otherwise the negative class. This makes it a binary classifier. Moreover, it can be implemented very easily and does not have critical hyperparameters to fine-tune. For this reason, it is widely used in clinical settings [27–29].

### Support vector machine

**Fig. 1:**
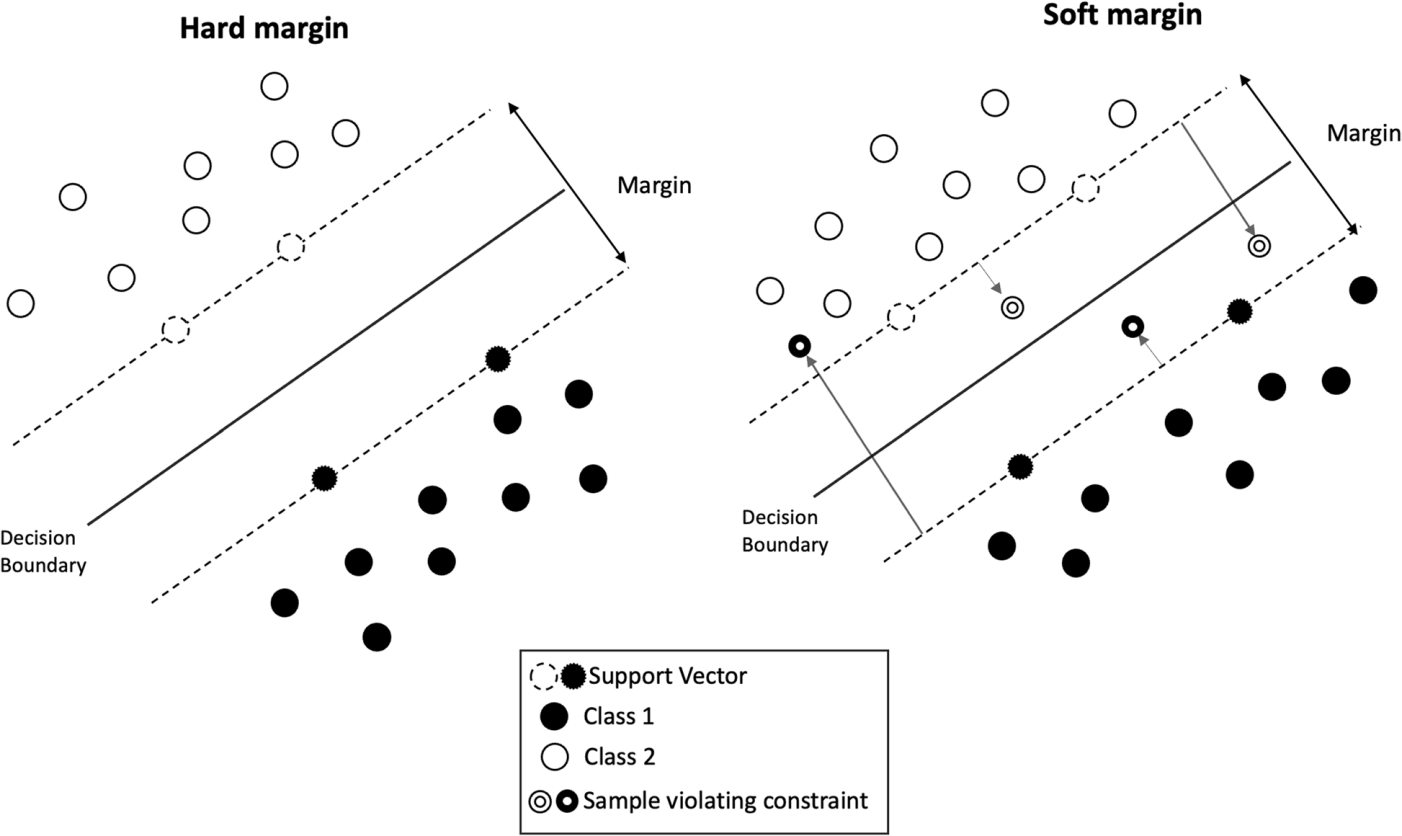
Graphical representation of hard and soft margin of a support vector machine. With the soft margin, some misclassifications (double circles) are allowed

The SVM [30] algorithm operates under the assumption that infinite hyperplanes can effectively separate data points. The primary objective of SVM is to identify the optimal hyperplane among this infinite set. The SVM algorithm considers some data more important than others for finding the best hyperplane: the support vectors. They are the samples (data points) most important to define the position and orientation of the best decision boundary (*i.e.,* the separating hyperplane). The distance between the separating hyperplane and the support vectors is called “margin.” The decision boundary that maximizes the margin is called “hard margin.” Sometimes, it is necessary to allow some classification error (misclassification) to improve the generalization capability: this is the main idea of the “soft margin”. All these elements can be seen in Fig. 1.

**Fig. 2:**
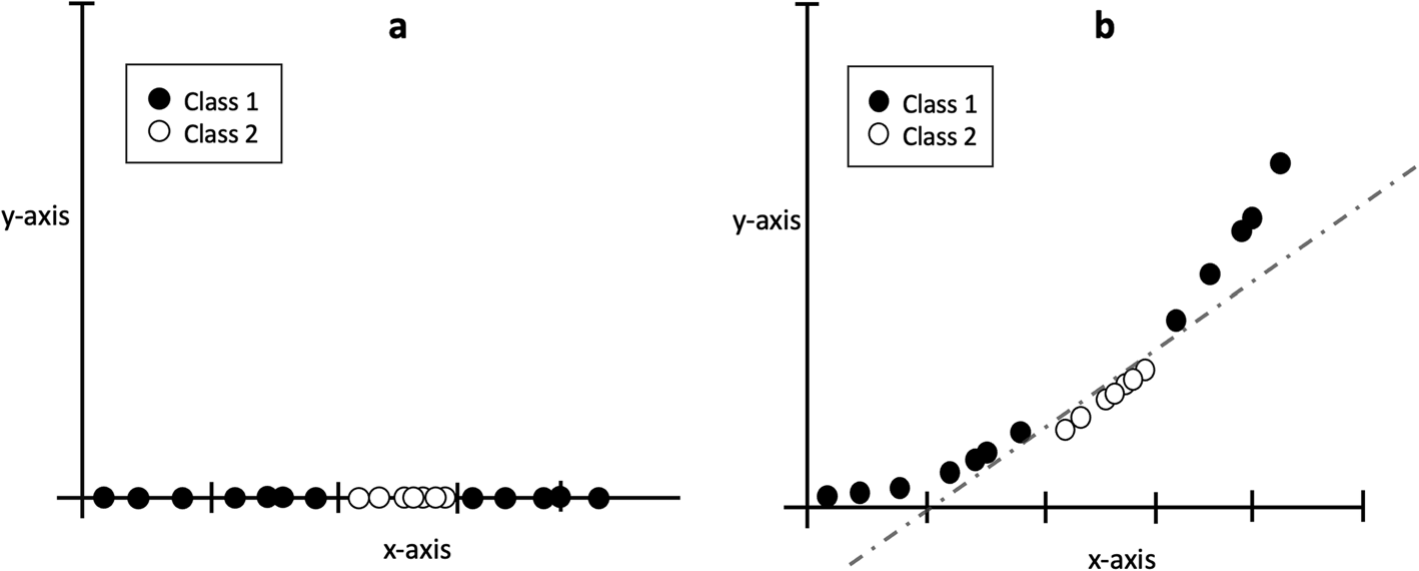
**a** The data on the *x*-axis are the original non-separable data. **b** Application of a second-degree polynomial function to make the two classes separable

To manage the trade-off between hard margin and soft margin, it is possible to use the regularization hyperparameter *C*. A small value of *C* causes greater misclassifications in training, resulting in a lower training performance but a higher generalization. Conversely, a high value of *C* minimizes the number of misclassified samples resulting in a high training performance but a lower generalization. In addition, in real scenarios, the data are not linearly separable as shown in the left panel of Fig. 2. In this case, the SVM algorithm uses kernels. Kernels are special functions applied to the original data, transforming it into a separable space. For example, as shown in the right panel of Fig. 2, a second-degree polynomial function can be applied to make the data separable. There are several types of kernels, and their choice can radically change the data distribution [31].

### Tree ensembles (TEs)

Ensemble learning, in general, employs a combination of various models to yield superior results compared to individual models [32]. It assumes that the combination of multiple weak learners results in a more robust and powerful learner. In the case of TE, the weak learners are the decision tree models [33, 34]. In this case, several DTs must be trained to build the TE. Despite the longer training time, the ensemble techniques result in improving overall accuracy. For this reason, random forest (RF) and gradient boosting (GB) are two of the most widely used SL algorithms for classification.

#### Random forest

**Fig. 3:**
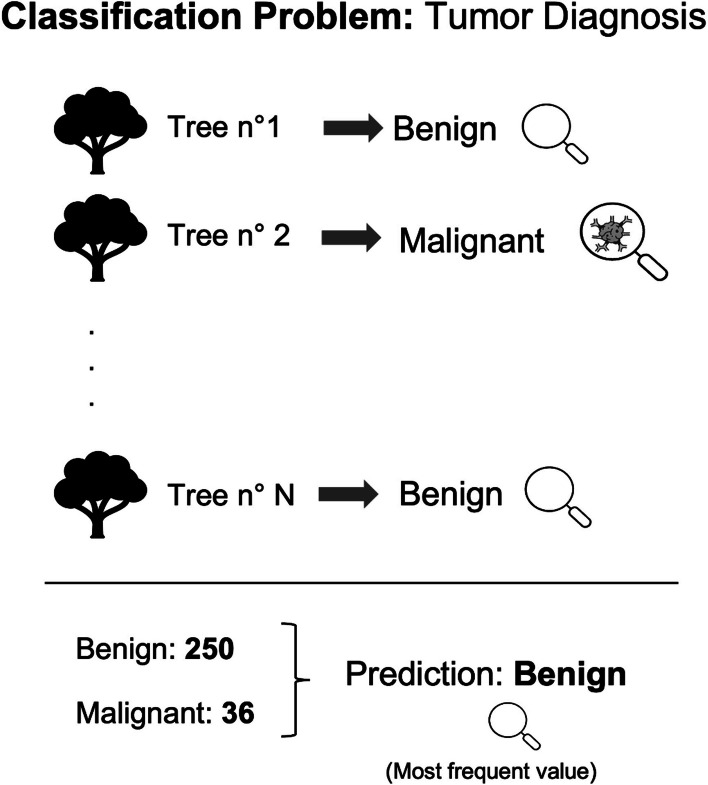
Application of the random forest algorithm. Each decision tree in the forest calculates its own prediction: 250 trees predicted the analyzed sample as benign and 36 as malignant. It is shown that the result is the most frequent prediction made by the entire forest (benign tumor)

The RF algorithm trains *n* DTs by considering a different random subset of the entire dataset for each DT [35]. For the generation of all subsets, RF uses a particular technique called *bagging*. *Bagging* is a meta-algorithm that allows training each DT considering only a random portion of the dataset (data and features) [36], creating vastly different results for each individual DT. This means that in a RF, there are DTs trained on different data and features, and each individual tree calculates its own prediction. The strength of RF lies in aggregating the predictions of all DTs through a voting mechanism, improving the stability and accuracy of the algorithm. An important hyperparameter to set is the number of estimators, *i.e.,* the number of DTs in the forest. There is no general rule for fixing the number of estimators [37]. Another parameter to manage is the maximum number of features used for training each DT. Typically, this value can be set as the square root of the total number of features. RF is a good choice in the case of missing data and noise data [38]. An example is shown in Fig. 3.

#### Gradient boosting

The GB algorithm uses DTs added sequentially to create the final model [39]. The main distinction between the RF and GB algorithms lies in the generation and aggregation of DTs. Specifically, in the GB algorithm, DTs are sequentially built to enhance the shortcomings of previously trained DTs. The primary goal of the training process in GB is to minimize a model’s loss function by iteratively introducing weak DT learners, thereby improving the subsequent DTs. This method is called boosting ensemble method. During the training process, more importance is provided to misclassified examples, and then, intuitively, new weak learners are added to focus on areas where existing learners perform poorly. At the end of the training, the result is a model that has exploited the weaknesses of the previous ones improving the generalization capabilities. An efficient and flexible implementation of the GB algorithm is provided by XGBoost [40], in which the training process is particularly fast [41].

### K-nearest neighbors

**Fig. 4:**
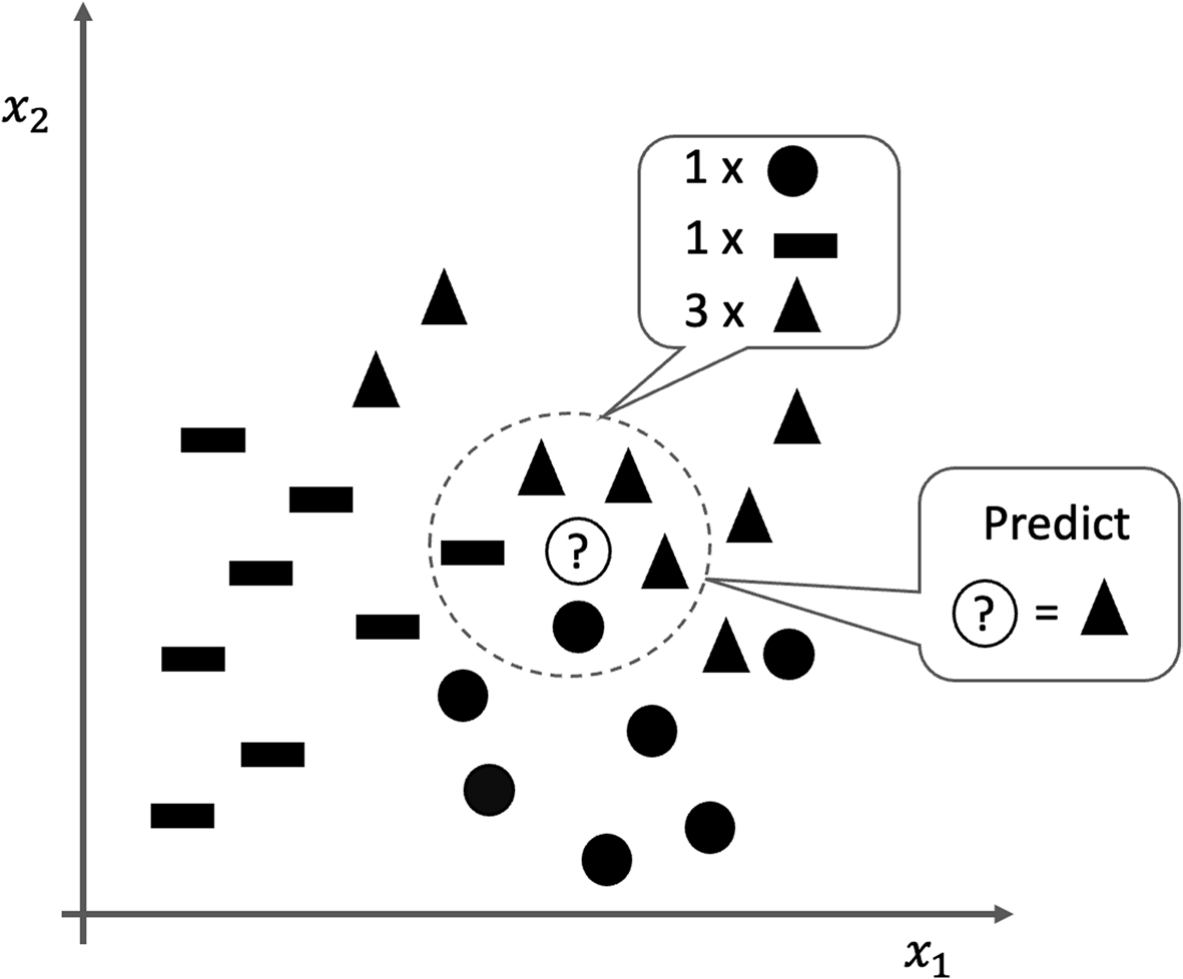
Representation of how the k-nearest neighbors algorithm works. Considering the new point to classify (?), the category is assigned based on the five nearest neighbors (*k* = 5). In this case, three triangles *versus* one circle *versus* one rectangle

K-nearest neighbors are one of the simplest classification methods in which the algorithm finds the *k*-nearest examples in the training set to assign the class of the new data. Figure 4 illustrates how this algorithm works. Specifically, a new data point denoted by the symbol “?” is classified as a “triangle” based on its five nearest neighbors (*k* = 5). The training process involves calculating distances between data points, and when new data are introduced, these distances need to be recalculated. The k-nearest neighbors algorithm requires the setting of three main hyperparameters: the neighborhood cardinality (*k*) which defines how many neighbors will be checked for class assignment, the metric to estimate the distance between neighboring points, and the weight function to assign a weight according to the distance [42]. The core of this classifier depends mainly on the choice of metric to calculate the distance between the tested examples and the training examples [43].

## Deep learning classifiers

DL algorithms are considered a specialization of ML [44], in which a substantial architectural difference is present: the depth. Deep NNs are composed of many layers of neurons allowing for the discovery of patterns on multiple levels of representation, implementing the concept of “feature hierarchies” or “features of features.” CNNs and transformers are nowadays the main choices for medical image analysis, as they simultaneously address the extraction of highly informative features and their classification.

### Deep neural networks fundamentals

**Fig. 5:**
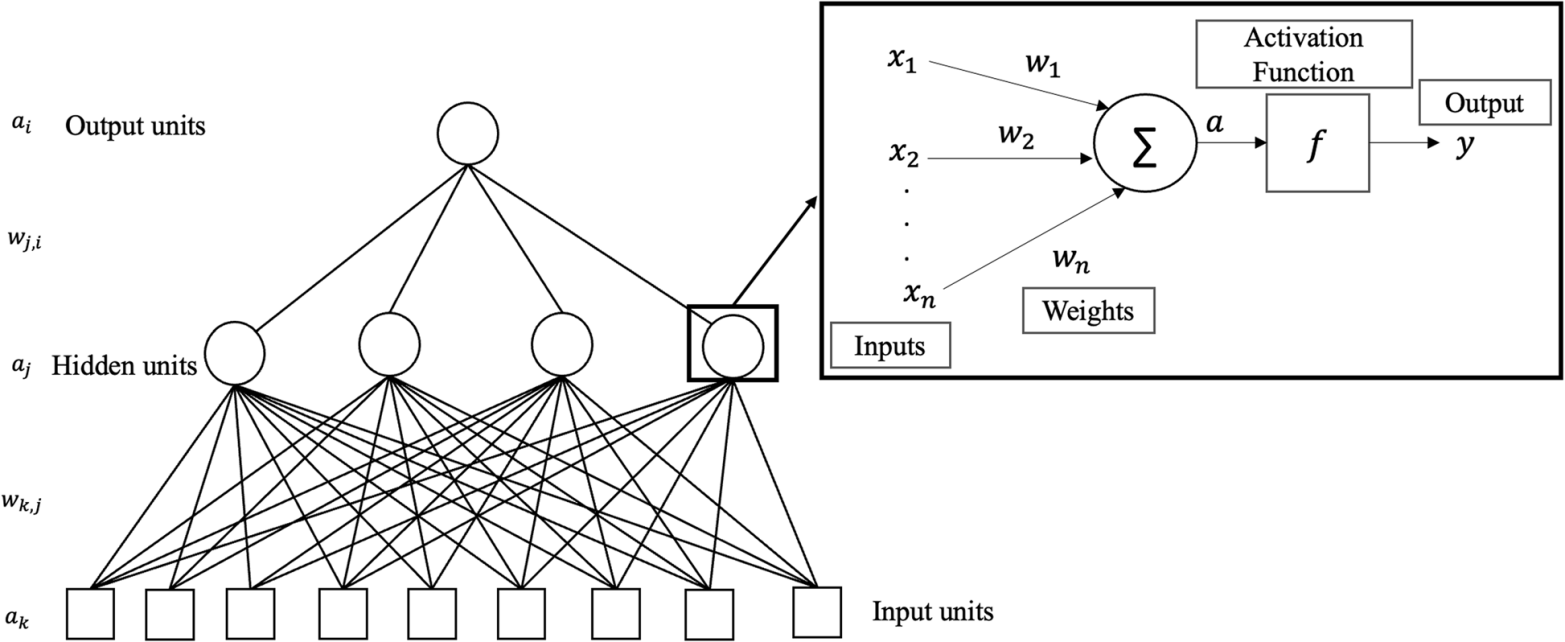
Representation of the multilayer perceptron, composed of one input layer, one hidden layer, and one output layer. Each individual unit of the hidden layer and output layer is a single perceptron, represented in the box

The upper-right box in Fig. 5 depicts the perceptron model proposed by McCulloch-Pitts in 1943 [45]. It can only handle linearly separable data and includes only an input layer and an output layer. To overcome this limitation, the concept of “depth” was introduced, giving rise to the multilayer perceptron (MLP) by adding several hidden layers. MLP is used for tabular data classification and is composed of fully connected layers, where each neuron calculates a weighted sum of inputs and applies an activation function to the result. During training, errors are computed through a loss function and propagated backward through a back-propagation mechanism. The optimizer is used for network weights updating. Setting the network hyperparameters [46, 47], such as number of layers, neurons, epochs, and learning rate, can be challenging and varies based on the specific task and dataset characteristics. There is no general rule for setting these hyperparameters. An interesting aspect lies in the depth of the architecture. Implementing very deep architectures may seem an excellent choice because it would improve the feature hierarchy extraction process. However, according to the universal approximation theorem, with only one hidden layer, NNs are universal approximators [48, 49].

### Convolutional neural networks

In the case of image analysis, the classifiers discussed in the previous section require that images or ROIs are described through features: these can be handcrafted features and represent the relationships between the gray levels, texture, or shape of a ROI [8, 9]. It is also possible to extract higher-level handcrafted features such as wavelet features, which showed remarkably interesting results in several tasks [50]. CNNs, conversely, include feature extraction in their workflow: given an input image or ROI, they extract the most informative features and then exploit these features for classification using the abovementioned MLP (often referred as “dense layers”) [10]. For this reason, CNNs are widely used in medical image analysis [51–53].

CNNs are a hot topic in research, leading to many complex architectures. They vary based on factors such as layer quantity, activation functions, and layer arrangement, leading to extensive discussions in the literature about CNN architecture. For this reason, we discuss only the general fundamentals behind the most popular CNN architectures (*e.g.,* Visual Geometry Group, ResNet, Inception [54]).

**Fig. 6:**
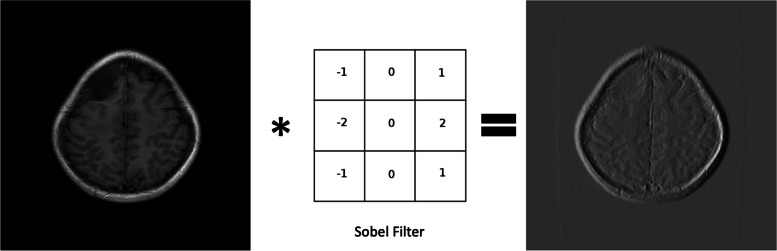
Example of convolutional operation between an input image (a T1-weighted magnetic resonance image of the brain) and the Sobel filter for edge detection

A CNN is composed of sequential layers, starting with the input layer representing an image as a matrix of pixels *width × height × channels* or in the case of three-dimensional images *width × height × depth × channels*. This is followed by the alternating of convolutional layers, pooling layers, or many other layers. Convolution involves applying a kernel (or filter) to the input image. In CNNs, these filter values are learned during training, allowing the network to determine their roles automatically (*e.g.,* filters for edge detection, blurring, noise reduction [55]). Figure 6 shows the result of convolution operation between the image and an edge detection filter. The kernel size is a hyperparameter to define a priori, as well as the activation function to apply after each convolutional layer. Images convolved with kernels return the so-called feature maps. To improve CNN performance and speed up training while reducing the number of learnable parameters, pooling layers are often used.

**Fig. 7:**
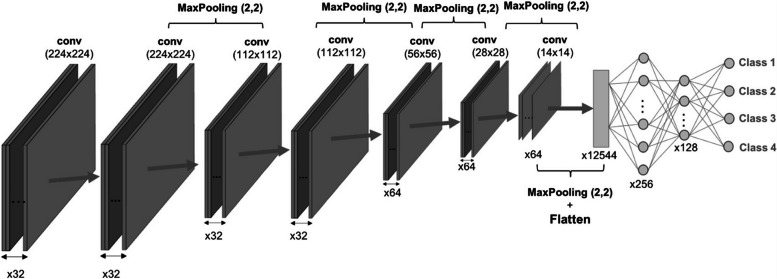
Example of convolutional neural network architecture. The input images are fed into the convolutional and pooling layers for feature extraction. In the end, the resulting flattened feature vector is fed into the dense layer to perform the classification task

There are several types of pooling layers, as discussed by Nirthika et al. [56]. The alternating of convolutional and pooling layers aids the network to focus on both low-level and high-level features. Early layers extract low-level features, while deeper layers capture more abstract high-level features, which are crucial for image classification. These extracted features are referred to as *deep features* or *learned features*. Finally, a MLP (dense layers) uses the resulting feature vector for the classification. Figure 7 shows an example of CNN, composed by a two-dimensional input image, several convolutional and pooling layer, a flattened layer to convert the feature maps into a feature vector, and eventually the MLP for classification.

### Vision transformers

A new frontier for image analysis lies in ViTs [57]. Transformers have been successfully applied to several computer vision problems, achieving state-of-the-art results and prompting researchers to reconsider the supremacy of CNNs as de facto operators [58]. In contrast to CNNs, ViTs are able to model the relationships among various small patches in the image. The *transformer block* assumes the image is divided into a sequence of patches, where each patch is flattened to a vector. These flattened image patches are used to create lower-dimensional linear embeddings and fed into a *transformer encoder*, composed by a *multi-head attention* to find local and global dependencies in the image. ViTs and CNNs have advantages and disadvantages, and it remains unclear which architecture is better. Therefore, much of the research is focusing on developing models combining transformer and CNN [59]. It has been shown that the introduction of a transformer block to convolutional networks can improve efficiency and overall accuracy [60].

### Transfer learning

The efficacy of NNs is intrinsically related to the availability of large databases. However, especially in medical scenarios, obtaining such datasets represents a challenge primarily due to the invasive and onerous nature of data annotation procedures. Transfer learning (TL) allows the use of existing large available databases (*source dataset*), enabling model tuning on very small proprietary databases (*target dataset*) [61]. The model trained on the source database can be used as a feature extractor and after is fine-tuned only the classification layers (the discussed MLP). It is also possible to retraining all network weights, taking advantage of the weights already optimized on the source dataset and achieving better convergence. TL shows considerable effectiveness, especially when the source and target datasets describe the same phenomenon and have similar data distributions. TL on a target database typically yields improved classification results, faster learning, and enhanced final classification [62].

## How to choose a classifier

The famous “no free lunch” theorem [63], in essence, states that the average performance of any pair of algorithms across all possible problems is identical. The implication is that the performance of some algorithms is identical to a completely naive algorithm, and it would be impossible to establish one algorithm better than another one. However, depending on the task, some algorithms are more recommendable than others under certain conditions [64].

### Task analysis

The first choice is driven by the intrinsic structure of the classification task and the features involved. All the algorithms presented in the previous section are not designed for both binary and multiclass classification. SVM, for example, is only used for binary classification, and therefore, it requires ad hoc strategies to implement multiclass classification (one-*versus*-rest or one-*versus*-one [65, 66]). The algorithms discussed previously are versatile and can handle both continuous and discrete features. However, in some cases, specific configurations may be necessary, such as using the Hamming distance for binary variables when a k-nearest neighbors algorithm is employed.

### Dataset size

As discussed in previous sections, the development of ML techniques is driven by the exponential growth of available data. Although there is no threshold establishing a minimum number of instances to train a ML algorithm, working with less than 50 instances makes the results highly questionable [25]. Some statistical analyses calculated the relationship between the number of features and training samples: for example, it was seen that for LR, a minimum of 10 to 15 samples per feature will produce reasonably stable estimates [67].

In general, when small datasets are available, it is preferable to use simple algorithms, such as LR or linear SVM. TE have proven also their worth for classification in small datasets [33, 68–70] and are the most used along with SVM [24, 71]. There is no established and recognized general rule that establishes the minimum size of a dataset for deep training. Generally, one refers to a “small” dataset without quantifying this definition [72]. For example, Sarker [73] states that when data volume is small, DL algorithms often perform poorly, while standard ML algorithms lie to improved performance. Many works deal with deep training even with a few hundred samples [74]. In general, this represents a challenge in training deep models using small datasets [75]. Training with a few hundred samples (*e.g.,* about 100) is also addressed without the use of TL [76]. In fact, in the last case, a cross-validation strategy is employed. In general, DL solutions are preferred when a lot of data are available [77]. Their use with small datasets is only justified if a large dataset is exploited for TL [78]. Alwosheel et al. [79] proposed a rule of thumb in which a minimum sample size of 50 times the number of weights in the network is required for training while a more conservative advises using at least ten times the number of weights.

### Explainability requirements

The significant insufficient transparency of ML algorithms represents a pivotal challenge for the integration of these systems into clinical practice. Usually, ML algorithms are denoted as black box, meaning that the inner workings of the models and their decision-making processes are not readily transparent or directly understandable. Fortunately, in recent years, *explainable AI* has emerged to address the problem of poor interpretability, to make the learned logic accessible and the process understandable by humans [80–83].

Algorithms such as DTs, and LR, are inherently interpretable, *i.e.,* it is possible to understand their decision-making process without the use of explainable AI methods. For this reason, these methods are preferred when few data are available, and simple and linear models are sufficient. Other SL algorithms such as TE are not inherently explainable, but several explainable AI methods can be employed for their global and local explanation [84].

A *global explanation* is important to understand the most important features that globally affect the predictions. Conversely, a *local explanation* focuses on elucidating the system’s decision for a particular instance, such as a patient. This approach allows for a detailed examination of the model’s findings and facilitates clinical validation and comparisons with existing medical literature [18]. These considerations carry significant ethical, legal, and trust-related implications. When intelligible inputs such as clinical, laboratory, or radiomic features are used, an explanation results to be straightforward. Conversely, learned features (for example, extracted via CNNs) are unintelligible. In the last case, explanations frequently are addressed considering the *saliency maps* computation. These maps highlight the regions within images that are most significant in the prediction process, thereby offering a form of local explanation [78]. Despite their widespread use, it has been demonstrated that saliency maps can yield inconsistent explanations [85, 86]. Consequently, SL solutions are often favored over DL approaches when explainability is mandatory.

### Available computing resources

The computing resources provided by current mid-range computers are suitable for training the discussed SL algorithms. For DL models, on the other hand, a high-performance graphics processing unit is required. In addition to performance, the graphics processing units must have a high amount of memory, especially when implementing architectures with several million of parameters. Some cloud computing services (*e.g.,* Google Colaboratory, https://colab.research.google.com/) are a good solution, especially for DL training for small/medium applications.

## Conclusions and future perspectives

This review discussed the main ML-based classifiers with educational purpose. SL models necessitate the presence of comprehensive disease-related features. In the context of medical images, these features may encompass radiomics (*radiomic features*) or be derived through NNs (*deep or learned features*). Following this feature extraction, the classification task is executed. DL architectures such as CNNs and ViTs integrate both feature extraction and classification within a unified pipeline. It is not possible to establish one algorithm or hyperparameter configuration better than others. However, some guidelines such as the task to be solved, the dataset size, the available computing resources, and the explainability requirements are important aspects to consider. While radiomic features provide a higher degree of interpretability, deep features are inherently more informative, thus enabling the creation of highly accurate models.

The model explanation is part of a more comprehensive concept, assuming central importance: *trustworthy AI* [87]. In sight of this, the conventional ML pipeline should be expanded with explainable methods to focus on ethical perspectives [88] and implement bias detection, fairness, and systems security, to comply with regulations such as the European General Data Protection Regulation (GDPR) [89], and, finally, to increase human-machine trust [90, 91].

## Data Availability

Not applicable.

## Abbreviations

CNN: Convolutional neural network
DT: Decision tree
DL: Deep learning
GB: Gradient boosting
LR: Logistic regression
ML: Machine learning
MLP: Multilayer perceptron
NN: Neural network
RF: Random forest
ROI: Region of interest
SL: Shallow learning
SVM: Support vector machine
TL: Transfer learning
TE: Tree ensemble
ViT: Vision transformer
